# The role of working conditions in educational differences in all-cause and ischemic heart disease mortality among Swedish men

**DOI:** 10.5271/sjweh.4158

**Published:** 2024-05-01

**Authors:** Melody Almroth, Tomas Hemmingsson, Daniel Falkstedt, Katarina Kjellberg, Emma Carlsson, Kuan-Yu Pan, Karin Berglund, Emelie Thern

**Affiliations:** 1 Institute of Environmental Medicine, Karolinska Institutet, Stockholm, Sweden.; 2 Department of Public Health Sciences, Stockholm University, Stockholm, Sweden.; 3 Centre for Occupational and Environmental Medicine, Region Stockholm, Stockholm, Sweden.; 4 Department of Global Public Health, Karolinska Institutet, Stockholm Sweden.

**Keywords:** cardiovascular disease, heavy physical workload, job control, social inequality

## Abstract

**Objectives:**

This study aims to investigate the extent to which low job control and heavy physical workload in middle age explain educational differences in all-cause and ischemic heart disease (IHD) mortality while accounting for important confounding factors.

**Methods:**

The study is based on a register-linked cohort of men who were conscripted into the Swedish military at around the age of 18 in 1969/1970 and were alive and registered in Sweden in 2005 (N=46 565). Cox proportional hazards regression models were built to estimate educational differences in all-cause and IHD mortality and the extent to which this was explained by physical workload and job control around age 55 by calculating the reduction in hazard ratio (HR) after adjustments. Indicators of health, health behavior, and other factors measured during conscription were accounted for.

**Results:**

We found a clear educational gradient for all-cause and IHD mortality (HR 2.07 and 2.47, respectively, for the lowest compared to the highest education level). A substantial part was explained by the differential distribution of the confounding factors. However, work-related factors, especially high physical workload, also played important explanatory roles.

**Conclusion:**

Even after accounting for earlier life factors, low job control and especially high physical workload seem to be important mechanistic factors in explaining educational inequalities in all-cause and IHD mortality. It is therefore important to find ways to reduce physical workload and increase job control in order to decrease inequalities in mortality.

The finding that higher education is related to better health has a long history in the human sciences. Relative educational differences in mortality have been consistently found in a wide variety of populations and subgroups (1, 2) and across birth cohorts (3). Ischemic heart disease (IHD) is the number one cause of death worldwide and education has also been found to be one of the important social determinants of IHD (4, 5).

While the Nordic countries have previously been recognized as having a relatively equal distribution of resources, mortality differences according to education level are still persistent (1). In fact, those with a university education are expected to live over five years longer than those with only compulsory education in Sweden, and this gap has grown over time (6). Large educational differences in incident IHD have also been found in Sweden (5).

The complex mechanisms linking education to health and mortality are not entirely understood. A recent systematic review identified education as one of the key social determinants of health regarding cardiovascular disease and acknowledged both its direct and indirect effects through other social determinants of health (4). There are several hypothesized indirect mechanisms, including physical and psychosocial working conditions (7).

Several systematic reviews and meta-analyses have determined that heavy physical workload is associated with all-cause mortality among men (8), that physical workload is potentially associated with IHD mortality (9), and that job control is the most consistent aspect of the psychosocial work environment related to both all-cause and cardiovascular mortality (10). These findings could possibly be explained by psychosocial stress leading to a negative physiological response (11) and physical strain on the cardiovascular system with too little time for recovery (9).

There is evidence that working conditions may play a mediating role in the relationship between socioeconomic position (SEP) and health (12), yet few high-quality studies have looked at the explanatory role of occupational exposures in relation to educational differences in mortality. One previous study from the United States investigated job complexity and hazardous working conditions as mediating factors on the pathway between education and all-cause mortality and found varying mediating patterns according to gender, race, and type of occupational factor (13). A Finnish study investigated a wide variety of physical and psychosocial occupational exposures in explaining socioeconomic differences in cardiovascular mortality and found lower job control and higher physical workload to be among the most important occupational exposures estimated (14). A Swedish study, on the other hand, found weak evidence for psychosocial occupational factors as an explanation of education differences in cardiovascular mortality (15). Physical occupational exposures have otherwise mostly been considered as explanatory factors for other outcomes such as sick leave or self-reported health (16, 17).

Adding to the complexity of understanding important mechanisms in educational differences in all-cause and IHD mortality, individual resources preceding education are also important to consider. This concept is known as indirect selection, where factors such as cognitive ability, mental and physical health, and personality may determine both educational and occupational achievement as well as later health factors (18). There is evidence that intelligence (19), SEP, and health behaviors (20) may play a particularly important role. Thus, it would be important to account for such factors in determining the explanatory role of working conditions in educational differences in all-cause and IHD mortality.

This study aimed to investigate the extent to which job control and heavy physical workload explain educational differences in all-cause and IHD mortality while accounting for earlier life factors representing early SEP measured in childhood as well as cognitive ability, and health and behavioral factors measured during late adolescence.

## Methods

### Study population

This study is based on a cohort of men who were conscripted into the Swedish military in 1969/1970. This cohort includes 50 087 men and has previously been described (21). We limited the population to those who had completed survey information during conscription and were alive and registered in Sweden in 2005 (N=46 565). We then linked the conscription information to the national registers in Sweden. The majority of the men were born between 1949 and 1951. Military service was obligatory for all men aged 18–20 years in Sweden during this time and covered at least 90% of the male population in this age group. Conscription involved a series of medical, physiological, and psychological testing using questionnaires, interviews, and physical tests (22).

**Figure 1:**
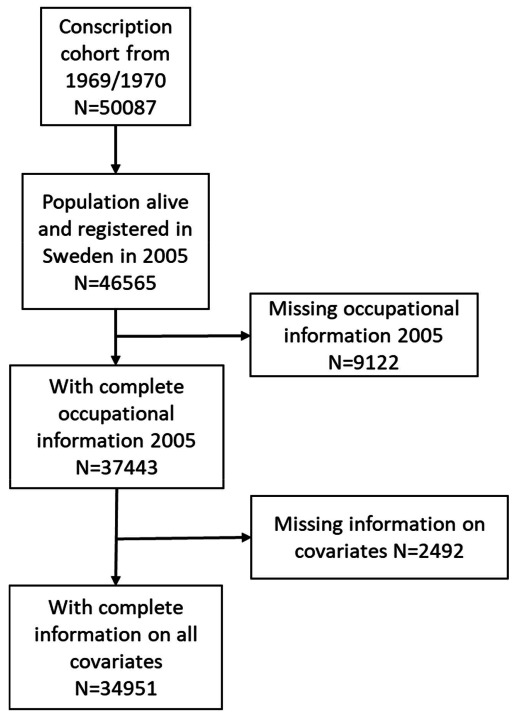
Selection of study sample.

Those who were missing information on their occupation in 2005 were excluded from the study (N=9122) as well as those with missing information on any other covariate (N=2492) The final analytical sample is 34 951 men. Figure 1 shows the flow of selection into the study.

Ethical approval was obtained from the Regional Ethics Review Board in Stockholm reference number 2017/1224-31, 2018/1675-32.

### Measures

*Education.* The highest attained education was taken from the Swedish Longitudinal Integrated Database for Health Insurance and Labor Market Studies (LISA register) in 2005 and was categorized from seven original categories into five based on total years of education: (i) <9 years includes compulsory education only (primary and lower secondary school); (ii) 10–11 years corresponds to ≤2 additional years of upper secondary school beyond compulsory school; (iii) 12 years corresponds to a completed 3-year upper secondary school education; (iv) 13–15 years corresponds to upper-secondary school plus ≤3 years of university education; and (v) >15 years corresponds to >3 years of university education.

*Mortality.* The cause of death register was used to identify all deaths (all-cause mortality) from 2006 until the end of 2020. The men were roughly aged 54–71 years during this time period. IHD mortality was identified using the International Classification of Disease version 10 (ICD-10) codes I20-I25 as either the underlying cause or contributing cause of death.

*Socioeconomic position (childhood).* SEP during childhood was measured through linkage of the index person to their biological or adoptive parents. Census information from the Swedish National Population and Housing Census was used to identify the occupation of the parents in 1960, when the index persons were around 9–11 years old. Occupational information was taken primarily from the father, though the mother’s occupation was used when this information was missing. This occupational information was then categorized according to Statistics Sweden’s recommended classification as unskilled manual workers, skilled manual workers, assistant non-manual workers, intermediate non-manual workers, higher non-manual workers, farmers, and those with no parental occupation reported (23).

*Conscription variables (late adolescence).* Several factors were chosen from the conscription register to capture important aspects of late adolescence that could hypothetically contribute to educational differences in all-cause and IHD mortality as well as to the potentially explanatory role of working conditions.

Divorced parents were identified through a questionnaire item.

The conscription evaluation included physical and psychiatric examinations, where a medical professional (physician and/or psychiatrist) recorded diagnoses. For this study, we identified psychiatric diagnoses (codes 290–315) and musculoskeletal disorders (MSD) diagnoses (codes 713–738) in the ICD-8 system.

Low emotional control includes a combined assessment of low stress tolerance, anxiety, reduced functioning due to psychosomatic symptoms, uncontrollable nervousness, and aggression.

Body mass index (BMI) was calculated based on height in centimeters and weight in kilograms. We categorized this based on the cutoff value of 25 kg/m^2^, where ≥25 kg/m^2^ is classified as overweight.

Both systolic and diastolic blood pressure were measured in mmHg (millimeters of mercury). Hypertension was defined as having a systolic blood pressure measure of ≥140 mmHg and a diastolic blood pressure measure of ≥80 mmHg.

Alcohol consumption was reported by the conscripts and categorized based on grams of 100% alcohol consumed per week as 0, 1–100, 101–250, or >250 grams. Smoking was also self-reported and categorized based on whether the person smoked ≥5 cigarettes per day or not.

Cognitive ability was assessed using tests for synonyms, induction, spatial capacity, and technical understanding. All tests began with more simple questions and problems and progressed in difficulty. The scores of the four tests were combined and categorized according to their standard nine (stanine) distribution. We further categorized this into low (categories 1–3), medium (categories 4–6), and high (categories 7–9).

*Working conditions (mid-50s).* Occupational physical workload and job control in 2005 were classified using job exposure matrices (JEM). The exposure level to physical workload and job control is measured at the occupational level and linked to the index person based on their registered occupation in 2005. The JEM are based on the Swedish Work Environment Surveys which were administered every second year from 1997 to 2013. The JEM are based on around 90 000 responses for 355 occupations.

Physical workload was measured through eight survey items regarding physical work that involves heavy lifting, uncomfortable working postures, repetitive work, and physically demanding work, as has been previously described (24). A gender-specific index mean value for these eight items was calculated to assess the occupational overall physical exposure. This value was then linked to individuals based on their registered occupation in 2005. These values were then categorized into quintiles based on the distribution of physical workload in the study population.

Job control was measured using the JEM for psychosocial exposures. It is measured based on four questions on decision authority and three questions on skill discretion. The questions measuring decision authority measure the individual’s ability to determine which tasks to do, the pace of the work, when to take breaks, and the structure of their work, which has also been previously described in more detail (25). The questions regarding skill discretion measure opportunities for learning and development, problem-solving, and training opportunities, which have also been previously described (26). Job control was also measured as a gender-specific mean score for each occupation. These values were then categorized into quintiles based on the distribution of job control in the study population. A validation of an older version of this JEM has been published (27).

### Statistical analysis

The distribution of covariates was explored according to level of education. Pearson’s chi-square tests were used to test descriptive differences in the study population. Cox proportional hazard regression models were used to estimate associations between each covariate and all-cause and IHD mortality respectively. These models estimate hazard ratios (HR) with 95% confidence intervals (CI). Cox proportional hazards regression models were also used to estimate educational differences in all-cause and IHD mortality. Follow-up time was calculated from 1 January 2006, until the date of death, migration from Sweden, or the end of follow up on 31 December 2020. The men were in their mid-to-late 50s during the start of follow-up and in their late 60s or early 70s at the end of follow-up. Age was used as the underlying time scale in all models.

A crude model was first used to estimate the HR comparing the risk of all-cause and IHD mortality according to the different education levels. Model 1 was adjusted for all early life factors and factors measured at conscription. This includes childhood SEP, divorced parents, psychiatric and MSD diagnoses at conscription, emotional control, BMI, blood pressure, alcohol consumption, smoking, and cognitive ability. Model 2 was adjusted for all of the covariates in model 1 plus physical workload in 2005. Model 3 was adjusted for all of the covariates in model 1 plus job control in 2005. Finally, model 4 was adjusted for all of the covariates in model 1 plus physical workload and job control.

Interaction terms between education and working conditions were added to multi-adjusted models and were not found to be significant, therefore main effects models are reported. Percent attenuation of the HR after the inclusion of each set of covariates was calculated to determine the extent that associations were explained by including these factors. The percentage of HR reduction was calculated using the formula [(HR_1_-HR_2_)/(HR_1_-1)]×100. Model 1 was compared to the crude model, while all subsequent models were compared to model 1. The 95% CI for the percentage of HR attenuation were obtained through bootstrap estimation using Efron’s quantile method performed with 1000 resamplings.

Additional models were also built as supplementary analyses to investigate specific combinations of variables not necessarily shown in the main analysis. This includes one model only adjusting for physical workload, one model only adjusting for job control, one model adjusting for all early life and conscription factors except cognitive ability, and one model additionally adding cognitive ability. All supplementary models were compared to the crude model.

All analyses were done using SAS Enterprise Guide version 8.3 (SAS Institute, Cary, NC, USA).

## Results

During the follow-up period, 3345 men died (9.6%), of which 721 deaths were due to IHD (2.1%).

Being a smoker and having parents in manual jobs or divorced parents, psychiatric or MSD diagnoses during conscription, low cognitive ability or emotional control, a BMI of ≥25 kg/m^2^, high blood pressure, and high alcohol consumption were all more common among men with lower education (table 1). Having a high physical workload and low job control were also much more common among lower-educated men. Additionally, the lower the education level, the higher the risk of all-cause and IHD mortality.

**Table 1:** Covariates according to level of education. [MSD=musculoskeletal disorder; BMI=body mass index; IHD=ischemic heart disease].

		>15 ^a^			13–14 ^b^			12 ^c^			10–11 ^d^			<9 ^e^		P-value ^f^
		N	%		N	%		N	%		N	%		N	%	
Total		6469	18.5		5363	15.3		5597	16.0		9797	28.0		7725	22.1	
Childhood socioeconomic position																<0.0001
	Unskilled manual	1118	17.3		1379	25.7		1646	29.4		3564	36.4		3174	41.1	
	Skilled manual	1000	15.5		1171	21.8		1363	24.4		2720	27.8		1932	25.0	
	Lower non-manual	1002	15.5		726	13.5		651	11.6		848	8.7		474	6.1	
	Intermediate non-manual	1830	28.3		1048	19.5		931	16.6		999	10.2		484	6.3	
	Higher non-manual	921	14.2		299	5.6		283	5.1		204	2.1		118	1.5	
	Farmer	398	6.2		560	10.4		505	9.0		1000	10.2		1160	15.0	
	Not classified	200	3.1		180	3.4		218	3.9		462	4.7		383	5.0	
Divorced parents		424	6.6		375	7.0		481	8.6		1113	11.4		894	11.6	<0.0001
Psychiatric diagnosis		478	7.4		361	6.7		427	7.6		1055	10.8		1033	13.4	<0.0001
MSD diagnosis		1081	16.7		859	16.0		821	14.7		1585	16.2		1317	17.0	0.0044
Cognitive ability																<0.0001
	Low	108	1.7		207	3.9		520	9.3		2104	21.5		2874	37.2	
	Medium	1963	30.3		2246	41.9		2965	53.0		5989	61.1		4152	53.7	
	High	4398	68.0		2910	54.3		2112	37.7		1704	17.4		699	9.0	
Low emotional control		1507	23.3		1226	22.9		1335	23.9		2833	28.9		2583	33.4	<0.0001
BMI >25 kg/m^2^		229	3.5		261	4.9		332	5.9		697	7.1		672	8.7	<0.0001
High blood pressure		567	8.8		500	9.3		494	8.8		911	9.3		857	11.1	<0.0001
Alcohol consumption																<0.0001
	Does not drink alcohol	475	7.3		342	6.4		280	5.0		517	5.3		468	6.1	
	1–100 grams per week	4905	75.8		4091	76.3		4140	74.0		6775	69.2		5271	68.2	
	101–250 grams per week	1002	15.5		850	15.8		1063	19.0		2151	22		1626	21.0	
	>250 grams per week	87	1.3		80	1.5		114	2.0		354	3.6		360	4.7	
Smoking >5 cigarettes/day		1860	28.8		1887	35.2		2464	44.0		5076	51.8		4362	56.5	<0.0001
Physical work 2005																<0.0001
	Low	3426	53.0		1497	27.9		973	17.4		763	7.8		308	4.0	
	Medium low	1811	28.0		1666	31.1		1368	24.4		1462	14.9		675	8.7	
	Medium	990	15.3		1570	29.3		1252	22.4		1985	20.3		1275	16.5	
	Medium high	185	2.9		373	7.0		1043	18.6		2718	27.7		2650	34.3	
	High	57	0.9		257	4.8		961	17.2		2869	29.3		2817	36.5	
Job control 2005																<0.0001
	Low	115	1.8		313	5.8		967	17.3		2518	25.7		3050	39.5	
	Medium low	831	12.8		1137	21.2		1034	18.5		2161	22.1		1844	23.9	
	Medium	938	14.5		591	11.0		1155	20.6		2560	26.1		1538	19.9	
	Medium high	1287	19.9		1816	33.9		1434	25.6		1747	17.8		896	11.6	
	High	3298	51.0		1506	28.1		1007	18.0		811	8.3		397	5.1	
All-cause mortality		410	6.3		424	7.9		477	8.5		1068	10.9		966	12.5	<0.0001
IHD mortality		77	1.2		78	1.5		104	1.9		243	2.5		219	2.8	<0.0001

^a^ >15 = ≥3 years of university. ^b^ 13–14 = <3 years of university. ^c^ 12 = 3 years of upper secondary school. ^d^ 10–11 = <3 years of upper secondary school. ^e^ <9 = compulsory school or less. ^f^ P-value corresponds to chi-square test.

Men with parental occupational class lower than higher non-manual, divorced parents, a psychiatric diagnosis during conscription, low emotional control, and high alcohol consumption had a greater risk of both all-cause and IHD mortality (table 2). Having low cognitive ability, a BMI of of ≥25 kg/m^2^, high blood pressure, and smoking ≥5 cigarettes per day were associated with an increased risk of all-cause mortality and an even greater risk of IHD mortality. Having an MSD diagnosis at conscription was associated with a slight increased risk in all-cause and IHD mortality, but this did not show statistical significance. Increasing levels of physical workload were associated with a higher risk of all-cause and IHD mortality, which showed a dose–response pattern except for the highest category. Lower levels of job control were increasingly associated with all-cause and IHD mortality in a dose–response pattern.

**Table 2:** Hazard ratios (HR) and 95% confidence intervals (CI) of covariates according to all-cause mortality and ischemic heart disease (IHD) mortality. [MSD=musculoskeletal disorder; BMI=body mass index].

		All-cause mortality		IHD mortality
		HR (95% CI)		HR (95% CI)
Childhood socioeconomic position				
	Unskilled manual	1.62 (1.35–1.94)		2.05 (1.35–3.11)
	Skilled manual	1.45 (1.21–1.75)		1.52 (0.99–2.33)
	Lower non-manual	1.32 (1.08–1.62)		1.22 (0.76–1.96)
	Intermediate non-manual	1.25 (1.02–1.52)		1.35 (0.86–2.12)
	Higher non-manual	1.00		1.00
	Farmer	1.07 (0.86–1.32)		1.15 (0.71–1.86)
	Not classified	1.35 (1.06–1.72)		1.93 (1.15–3.23)
Divorced parents				
	Not divorced	1.00		1.00
	Divorced	1.27 (1.14–1.41)		1.34 (1.07–1.68)
Psychiatric diagnosis				
	Yes	1.39 (1.25–1.54)		1.31 (1.05–1.64)
	No	1.00		1.00
MSD diagnosis				
	Yes	1.09 (0.99–1.19)		1.20 (0.99–1.45)
	No	1.00		1.00
Cognitive ability				
	Low	1.74 (1.58–1.92)		1.97 (1.61–2.42)
	Medium	1.22 (1.13–1.33)		1.26 (1.06–1.50)
	High	1.00		1.00
Emotional control				
	Low	1.27 (1.18–1.37)		1.20 (1.02–1.40)
	Normal-high	1.00		1.00
BMI (kg/m^2^)				
	<25	1.00		1.00
	>25	1.53 (1.36–1.72)		2.29 (1.84–2.84)
Blood pressure				
	High	1.12 (1.00–1.25)		1.53 (1.24–1.89)
	Normal	1.00		1.00
Alcohol consumption (grams)				
	0	0.89 (0.76–1.04)		1.09 (0.80–1.48)
	1–100	1.00		1.00
	101–250	1.29 (1.19–1.40)		1.12 (0.93–1.35)
	>250	1.85 (1.58–2.17)		1.89 (1.34–2.64)
Smoking (cigarettes/day)				
	>5	1.67 (1.56–1.79)		2.01 (1.73–2.33)
	<5	1.00		1.00
Physical work 2005				
	Low	1.00		1.00
	Medium low	1.23 (1.09–1.39)		1.43 (1.07–1.90)
	Medium	1.42 (1.26–1.60)		1.69 (1.28–2.23)
	Medium high	1.87 (1.67–2.10)		2.53 (1.95–3.28)
	High	1.80 (1.60–2.02)		2.51 (1.93–3.25)
Job control 2005				
	Low	1.93 (1.72–2.15)		2.59 (2.02–3.33)
	Medium low	1.63 (1.46–1.83)		2.04 (1.58–2.65)
	Medium	1.38 (1.22–1.55)		1.57 (1.20–2.06)
	Medium high	1.13 (1.00–1.28)		1.26 (0.95–1.66)
	High	1.00		1.00

Men with lower levels of education had a greater risk of all-cause mortality during the follow-up period, and there was a gradient in the association. HR ranged from 1.22 (95% CI 1.07–1.40) among men with 13–14 compared to ≥15 years of education to 2.07 (95% CI 1.85–2.33) for men with ≤9 compared to ≥15 years of education (crude model) (table 3). Adjusting for childhood SEP, divorced parents, psychiatric and MSD diagnoses, emotional control, BMI, blood pressure, alcohol consumption, smoking, and cognitive ability explained 39.5% (95% CI 31.4–48.6) of the associations among the lowest educated (model 1). Adjusting for physical workload in addition to these early life factors additionally explained 33.3% (95% CI 22.6–44.8) of the association between education and all-cause mortality in the lowest educated group beyond what was adjusted for in model 1 (model 2), while adjusting for the covariates in model 1 plus job control additionally explained 26.9% (95% CI 19.1–36.1) of the association between education and all-cause mortality among the lowest educated men, beyond the covariates included in model 1 (model 3). Adjusting for all covariates explained 31.6% (95% CI 21.0–42.9) of inequalities in all-cause mortality beyond the confounders adjusted for in model 1 among the lowest educated men.

**Table 3:** Crude and adjusted hazard ratios and 95% confidence intervals for associations between education level and all-cause mortality. [R=reduction.]

	>15		13–14			12			10–11			<9	
	HR (95% CI)		HR (95% CI)	% reduction (95% CI)		HR (95% CI)	% reduction (95% CI)		HR (95% CI)	% reduction (95% CI)		HR (95% CI)	% reduction (95% CI)
Crude	1.00		1.22 (1.07–1.40)			1.36 (1.19–1.55)			1.79 (1.60–2.01)			2.07 (1.85–2.33)	
Model 1 ^a^	1.00		1.18 (1.03–1.35)	19.2 (9.1–44.7)		1.23 (1.08–1.41)	34.1 (23.3–53.2)		1.51 (1.33–1.71)	35.4 (26.9–45.0)		1.65 (1.44–1.88)	39.5 (31.4–48.6)
Model 2 ^b^	1.00		1.13 (0.98–1.30)	28.8 (14.6–72.8)		1.13 (0.98–1.30)	45.5 (27.6–90.5)		1.33 (1.16–1.53)	34.7 (23.3–48.0)		1.43 (1.24–1.66)	33.3 (22.6–44.8)
Model 3 ^c^	1.00		1.15 (1.00–1.32)	15.6 (4.5–44.2)		1.17 (1.01–1.34)	28.8 (15.8–56.6)		1.39 (1.22–1.59)	23.9 (15.4–33.2)		1.47 (1.28–1.70)	26.9 (19.1–36.1)
Model 4 ^d^	1.00		1.14 (0.99–1.32)	19.3 (4.6–54.0)		1.15 (1.00–1.33)	36.0 (19.4–71.9)		1.36 (1.19–1.56)	28.9 (18.1–41.8)		1.44 (1.25–1.67)	31.6 (21.0–42.9)

^a^ Model 1 is adjusted for childhood socioeconomic position, divorced parents, conscription psychiatric diagnosis, conscription musculoskeletal diagnosis, emotional control, body mass index, blood pressure, alcohol consumption, smoking and cognitive ability. ^b^ Model 2 is adjusted for covariates in model 1 + physical workload in 2005. ^c^ Model 3 is adjusted for covariates in model 1 + job control in 2005. ^d^ Model 4 is adjusted for covariates in model 1 + physical workload and job control in 2005.

**Table 4:** Crude and adjusted hazard ratios and 95% confidence intervals for associations between education level and ischemic heart disease mortality.

	>15		13–14			12			10–11			<9	
	HR (95% CI)		HR (95% CI)	% reduction (95% CI)		HR (95% CI)	% reduction (95% CI)		HR (95% CI)	% reduction (95% CI)		HR (95% CI)	% reduction (95% CI)
Crude	1.00		1.21 (0.88–1.65)			1.57 (1.17–2.11)			2.15 (1.66–2.77)			2.47 (1.90–3.20)	
Model 1 ^a^	1.00		1.13 (0.82–1.55)	38.3 (–199.1–321.4)		1.36 (1.00–1.85)	36.5 (18.9–70.3)		1.68 (1.27–2.23)	40.5 (24.0–57.1)		1.75 (1.30–2.36)	48.7 (32.6–65.9)
Model 2 ^b^	1.00		1.04 (0.75–1.43)	72.7 (–653.2–651.1)		1.14 (0.83–1.57)	60.6 (31.9–210.9)		1.32 (0.98–1.80)	52.5 (33.7–86.6)		1.34 (0.97–1.86)	54.4 (35.8–86.0)
Model 3 ^c^	1.00		1.07 (0.77–1.48)	45.7 (–214.6–526.1)		1.23 (0.90–1.68)	36.8 (16.6–129.5)		1.45 (1.08–1.96)	33.4 (19.3–55.6)		1.45 (1.06–1.99)	39.9 (25.1–63.7)
Model 4 ^d^	1.00		1.05 (0.76–1.45)	63.0 (–539.0–700.0)		1.17 (0.85–1.60)	54.2 (27.2–246.6)		1.35 (1.00–1.84)	48.0 (29.1–81.1)		1.34 (0.97–1.86)	54.3 (34.7–87.3)

^a^ Model 1 is adjusted for childhood socioeconomic position, divorced parents, conscription psychiatric diagnosis, conscription musculoskeletal diagnosis, emotional control, body mass index, blood pressure, alcohol consumption, smoking, and cognitive ability. ^b^ Model 2 is adjusted for covariates in model 1 + physical workload in 2005. ^c^ Model 3 is adjusted for covariates in model 1 + job control in 2005. ^d^ Model 4 is adjusted for covariates in model 1 + physical workload and job control in 2005.

Men with lower levels of education were also more likely to die of IHD, and this risk increased as the level of education decreased (a gradient). The HR ranged from 1.21 (95% CI 0.88–1.65) for men with 13–14 compared to ≥15 years of education to 2.47 (95% CI 1.90–3.20) for men with ≤9 compared to ≥15 years of education. Adjusting for the factors measured in childhood and late adolescence explained 48.7% (95% CI 32.6–65.9) of the association between education level and IHD mortality among the lowest educated men. The additional adjustment for physical workload explained 54.4% (95% CI 35.8–86.0) of associations among the lowest educated men, while adjusting for all of the covariates from childhood and late adolescence plus job control explained 39.9% (25.1–63.7) of associations beyond the factors included in model 1. Finally, when adjusting for all covariates, 54.3% (95% CI 34.7–87.3) of the associations were explained beyond the factors included in model 1.

In further stepwise analyses, only adjusting for physical workload explained 35.0% (95% CI 26.4–43.7) of the associations between education and all-cause mortality and 51.6% (95% 38.7–66.0) for IHD mortality, while only adjusting for job control explained 32.2% (95% CI 25.3–39.1) for all-cause and 43.1% (95% CI 31.4–55.8) for IHD mortality among the lowest educated men (supplementary material, www.sjweh.fi/article/4158, tables S1 and S2). Adding cognitive ability in a separate step contributed to slightly larger attenuations compared to the model with all other factors from youth among the lower educated for both all-cause and IHD mortality.

## Discussion

Our results confirmed a clear educational gradient for all-cause and IHD mortality. Adjusting for SEP, health, and behavioral factors during childhood and late adolescence explained some of this association. Additionally adjusting for physical workload and job control showed further attenuations, though this was greater for physical workload.

The most relevant previous studies presented results which were somewhat in line with the present study. One study from the United States found that lower workplace hazards and higher job complexity mediated the association between education level and all-cause mortality among certain groups of individuals based on race and sex (13). This study, however, is difficult to directly compare with, as their focus was primarily on racial and sex differences and the present study only included Swedish men. A previous study from Finland identified job control and physical workload as important work-related factors but concluded that occupational factors only explained a small portion of inequalities in cardiovascular mortality (14). Though this study had some similarities in population and measures, the study is >20 years old and may reflect some differences in time and context. A previous Swedish study found that most of the educational differences in cardiovascular mortality were explained by conventional risk factors such as BMI and smoking rather than psychosocial work environment factors (15). Several studies investigating cardiovascular morbidity have also found psychosocial factors to be an important explanation of socioeconomic differences (28, 29).

The finding that physical workload appears to play an even more important role than job control is in line with a previous Swedish literature review and report which found more evidence for physical workload as an explanation of social inequalities in a variety of health outcomes (17). We found support for this considering both all-cause and IHD mortality. Physical work may be health damaging in several different ways leading to both higher cardiovascular risk and other health risks. This can include insufficient time to recover from prolonged strenuous work leading to increased heart rate, increased blood pressure, and inflammation (8). Those in heavy physical work may also have poorer health behaviors and worse socioeconomic resources leading to poorer health (further mediating factors). On the other hand, physical workload may also correlate with other dangerous occupational exposures including exposure to chemicals and particles (30), as well as fatal occupational accidents (31).

Our finding that SEP, health, and health behavior factors measured during childhood and late adolescence were important factors in explaining educational differences in all-cause and cardiovascular mortality is in line with previous studies that have identified many of these as important risk factors (20). Our study makes an important contribution to the literature by accounting for these factors which are likely to precede educational achievement and thus be important for selection into education and occupation as well as for later health and mortality. That working conditions still played a substantial role beyond these early factors points to the additional importance of occupational exposures.

Increasing inequalities in mortality may reflect the health benefits of the higher socioeconomic classes where gains in health are not seen to the same extent in the lower classes (32). Future studies should look at further explanatory mechanisms between heavy physical workload and low job control and mortality and investigate specific aspects of these occupational exposures that could be reduced in order to improve health and reduce inequalities. This may be particularly important considering that there is evidence that the educational gap in mortality has widened in birth cohorts over time, and that these inequalities are likely to continue to grow (33).

### Strengths and limitations

This study has several important strengths including extensive information throughout the life course collected in registers and at military conscription. The inclusion of all men who were conscripted into the military and using register data for follow-up prevents issues of selection and attrition bias. This also allows for a long follow-up time and a life-course perspective with regards to the data and adjustments. This includes the possibility of adjusting for a variety of factors that may predict selection into the labor market and later health outcomes. The use of education as a measure of social inequalities is also a strength because it is usually established during childhood and late adolescence and is less likely to represent health selection (where health would determine socioeconomic status rather than the other way around) compared with measures established later in life (34).

Several important limitations should also be mentioned. One major one is that this study only included men. However, IHD in this age group is expected to be more common among men (35). We used several items measuring job control and physical workload, but these factors only represent certain aspects of the physical and psychosocial work environment. The use of JEM is likely to result in non-differential misclassification, as they represent occupational and not individual levels of exposures. This could lead to an underestimation of the role of working conditions. Additionally, occupational exposures were only measured at one point in time and may not be representative of the entire working life. We have, however, found occupations to be quite stable over time (26, 36). Furthermore, because both job control and physical workload tend to be highly correlated (24), it remains difficult to completely disentangle their effects.

Though we were able to include measures of smoking and alcohol consumption during conscription, we did not have information on these behavioral patterns later in life. We were able to account for many earlier life risk factors, but we could not measure stressful life events outside of the working life relating to home and family life. Such factors may also play important explanatory roles in inequalities in mortality. One previous study found that personality played an important role in educational gradients in mortality among men (37). We were able to control for cognitive ability and emotional control, but there may be other important personality traits that we could not account for. Finally, the use of HR has limitations when it comes to analyses of mediation. HR provide a single point estimate for associations which could be time varying including time varying relationships between exposures, mediators, and outcomes. Methodological studies have aimed to estimate such structural aspects in mediation analysis, to strengthen causal interpretations (38).

### Concluding remarks

Physical workload and job control appeared to be mechanistic factors in explaining educational inequalities in all-cause and IHD mortality even when a variety of previous risk factors were accounted for. However, physical workload seems to be particularly relevant. With these findings in mind, it is important to find ways to improve both physical and psychosocial working conditions in order to prevent inequalities in mortality.

## Supplementary material

Supplementary material
