# Supplementary material for: The role of working conditions in educational differences in all-cause and ischemic heart disease mortality among Swedish men
Source: Scand J Work Environ Health. 2024 Apr 29;50(4):300–9. doi: 10.5271/sjweh.4158 (PMC11130708; doi:10.5271/sjweh.4158)
Supplement: Supplementary material [file SJWEH-50-300-S001.pdf]

The role of working conditions in educational differences in all-cause and ischemic heart disease mortality among Swedish men[1]

by Melody Almroth, PhD,[2] Tomas Hemmingsson, PhD, Daniel Falkstedt, PhD, Katarina Kjellberg, PhD, Emma Carlsson, MPh, Kuan-Yu Pan, PhD, Karin Berglund, Mpt, Emelie Thern, PhD

1. Supplementary tables
2. Correspondence to: Melody Almroth, Karolinska Institutet, Solnavägen 4, floor 10, 113 65, Stockholm, Sweden. [E-mail:melody.almroth@ki.se]

Table S1 Crude and adjusted hazard ratios and 95% confidence intervals for associations between education level and all-cause mortality (stepwise adjustments)

	≥15	13-14		12		10-11		≤9	
	**HR**	**HR (95% CI)**	**% reduction (95% CI)**	**HR (95% CI)**	**% reduction (95% CI)**	**HR (95% CI)**	**% reduction (95% CI)**	**HR (95% CI)**	**% reduction (95% CI)**
**Crude**	1.00	1.22 (1.07-1.40)		1.36 (1.19-1.55)		1.79 (1.60-2.01)		2.07 (1.85-2.33)	
**Model 1 (phys)**	1.00	1.15 (1.00-1.32)	31.8 (18.6-66.6)	1.20 (1.05-1.38)	42.6 (29.2-64.5)	1.51 (1.33-1.72)	35.4 (26.5-44.9)	1.70 (1.48-1.94)	35.0 (26.4-43.7)
**Model 2 (control)**	1.00	1.17 (1.02-1.35)	21.6 (10.6-42.1)	1.24 (1.08-1.42)	31.8 (21.0-46.7)	1.57 (1.38-1.77)	28.4 (21.0-35.8)	1.73 (1.52-1.96)	32.2 (25.3-39.1)
**Model 3 (phys + control)**	1.00	1.17 (1.02-1.34)	23.9 (10.2-52.1)	1.23 (1.07-1.41)	36.2 (23.2-58.3)	1.54 (1.35-1.75)	31.6 (22.6-41.8)	1.70 (1.48-1.94)	35.1 (26.4-44.3)
**Model 4 (youth)**	1.00	1.18 (1.03-1.35)	19.6 (10.0-42.7)	1.24 (1.09-1.42)	31.9 (22.7-48.0)	1.55 (1.37-1.74)	30.9 (24.7-37.9)	1.74 (1.53-1.97)	31.3 (25.6-37.5)
**Model 5 (youth + IQ)**	1.00	1.18 (1.03-1.35)	19.2 (8.0-41.7)	1.23 (1.08-1.41)	34.1 (22.5-53.2)	1.51 (1.33-1.71)	35.4 (26.8-44.4)	1.65 (1.44-1.88)	39.5 (31.3-48.1)
**Model 6 (youth + IQ + phys)**	1.00	1.13 (0.98-1.30)	42.5 (24.2-86.0)	1.13 (0.98-1.30)	64.1 (46.5-92.7)	1.33 (1.16-1.53)	57.8 (46.4-70.0)	1.43 (1.24-1.66)	59.7 (49.5-69.6)
**Model 7 (youth+ IQ + control)**	1.00	1.15 (1.00-1.32)	31.8 (15.9-63.4)	1.17 (1.01-1.34)	53.1 (37.3-79.5)	1.39 (1.22-1.59)	50.8 (41.2-62.1)	1.47 (1.28-1.70)	55.8 (46.3-65.8)
**Model 8 full model**	1.00	1.14 (0.99-1.32)	34.8 (17.9-79.0)	1.15 (1.00-1.33)	57.8 (40.9-86.9)	1.36 (1.19-1.56)	54.0 (42.9-66.5)	1.44 (1.25-1.67)	58.6 (48.7-69.3)

Model 1 is adjusted for physical workload.
Model 2 is adjusted for job control.
Model 3 is adjusted for physical workload and job control.
Model 4 is adjusted for childhood socioeconomic position, divorced parents, conscription psychiatric diagnosis, conscription musculoskeletal diagnosis, emotional control, body mass index, blood pressure, alcohol consumption, and smoking.
Model 5 is adjusted for covariates in model 4 + cognitive ability.
Model 6 is adjusted for covariates in model 5 + physical workload in 2005.
Model 7 is adjusted for covariates in model 5 + job control in 2005.
Model 8 is adjusted for covariates in model 5 + physical workload and job control in 2005.

Table S2 Crude and adjusted hazard ratios and 95% confidence intervals for associations between education level and ischemic heart disease mortality (stepwise adjustments)

	≥15	13-14		12		10-11		≤9	
	**HR**	**HR (95% CI)**	**% reduction (95% CI)**	**HR (95% CI)**	**% reduction (95% CI)**	**HR (95% CI)**	**% reduction (95% CI)**	**HR (95% CI)**	**% reduction (95% CI)**
**Crude**	1.00	1.21 (0.88-1.65)		1.57 (1.17-2.11)		2.15 (1.66-2.77)		2.47 (1.90-3.20)	
**Model 1 (phys)**	1.00	1.08 (0.78-1.49)	61.3 (-385.5-437.1)	1.26 (0.92-1.71)	55.2 (34.6-106.4)	1.57 (1.17-2.09)	50.7 (36.1-66.2)	1.71 (1.27-2.31)	51.6 (38.7-66.0)
**Model 2 (control)**	1.00	1.12 (0.81-1.54)	42.9 (-265.2-280.4)	1.35 (0.99-1.83)	38.7 (21.5-74.3)	1.72 (1.30-2.26)	37.4 (25.3-51.3)	1.84 (1.38-2.44)	43.1 (31.4-55.8)
**Model 3 (phys + control)**	1.00	1.10 (0.79-1.51)	53.4 (-246.4-347.6)	1.28 (0.94-1.75)	50.5 (31.3-88.7)	1.60 (1.20-2.14)	47.5 (33.5-64.1)	1.70 (1.25-2.30)	52.7 (39.5-67.0)
**Model 4 (youth)**	1.00	1.12 (0.82-1.54)	41.0 (-235.6-242.3)	1.36 (1.01-1.83)	37.0 (23.4-67.4)	1.70 (1.30-2.23)	38.8 (28.0-52.5)	1.84 (1.39-2.42)	43.2 (32.8-56.7)
**Model 5 (youth + IQ)**	1.00	1.13 (0.82-1.55)	38.3 (-152.3-256.0)	1.36 (1.00-1.85)	36.5 (19.0-71.2)	1.68 (1.27-2.23)	40.5 (25.3-58.5)	1.75 (1.30-2.36)	48.7 (33.0-65.7)
**Model 6 (youth + IQ + phys)**	1.00	1.04 (0.75-1.43)	83.1 (-613.1-636.2)	1.14 (0.83-1.57)	75.0 (50.1-138.3)	1.32 (0.98-1.80)	71.7 (55.5-92.8)	1.34 (0.97-1.86)	76.6 (61.1-93.1)
**Model 7 (youth+ IQ + control)**	1.00	1.07 (0.77-1.48)	66.5 (-259.4-459.2)	1.23 (0.90-1.68)	59.9 (37.6-113.0)	1.45 (1.08-1.96)	60.4 (44.0-80.7)	1.45 (1.06-1.99)	69.2 (53.3-86.2)
**Model 8 full model**	1.00	1.05 (0.76-1.45)	77.2 (-383.0-546.1)	1.17 (0.85-1.60)	70.9 (47.6-127.1)	1.35 (1.00-1.84)	69.1 (52.0-90.0)	1.34 (0.97-1.86)	76.6 (60.7-94.3)

Model 1 is adjusted for physical workload.
Model 2 is adjusted for job control.
Model 3 is adjusted for physical workload and job control.
Model 4 is adjusted for childhood socioeconomic position, divorced parents, conscription psychiatric diagnosis, conscription musculoskeletal diagnosis, emotional control, body mass index, blood pressure, alcohol consumption, and smoking.
Model 5 is adjusted for covariates in model 4 + cognitive ability.
Model 6 is adjusted for covariates in model 5 + physical workload in 2005.
Model 7 is adjusted for covariates in model 5 + job control in 2005.
Model 8 is adjusted for covariates in model 5 + physical workload and job control in 2005.
